# Interleukin-8 is the single most up-regulated gene in whole genome profiling of *H. pylori *exposed gastric epithelial cells

**DOI:** 10.1186/1471-2180-12-9

**Published:** 2012-01-17

**Authors:** Lars L Eftang, Ying Esbensen, Tone M Tannæs, Ida RK Bukholm, Geir Bukholm

**Affiliations:** 1 Department of Clinical Molecular Biology (Epigen), Institute of Clinical Medicine, University of Oslo, Akershus University Hospital, Lørenskog, Norway; 2 Department of Gastroenterological Surgery, Akershus University Hospital, Lørenskog, Norway; 3 Department of Clinical Molecular Biology (Epigen), Akershus University Hospital, Lørenskog, Norway; 4 Institute of Clinical Medicine, Akershus University Hospital, University of Oslo, Lørenskog, Norway; 5 Institute of Health and Society, University of Oslo, Oslo, Norway

## Abstract

**Background:**

The association between *Helicobacter pylori *infection and upper gastrointestinal disease is well established. However, only a small fraction of *H. pylori *carriers develop disease, and there are great geographical differences in disease penetrance. The explanation to this enigma lies in the interaction between the bacterium and the host. *H. pylori *Outer Membrane Phospholipase A (OMPLA) has been suggested to play a role in the virulence of this bacterium. The aim of this study was to profile the most significant cellular pathways and biological processes affected in gastric epithelial cells during 24 h of *H. pylori *exposure, and to study the inflammatory response to OMPLA^+ ^and OMPLA^- ^*H. pylori *variants.

**Results:**

Interleukin-8 was the most significantly up-regulated gene and appears to play a paramount role in the epithelial cell response to *H. pylori *infection and in the pathological processes leading to gastric disease. MAPK and NF-kappaB cellular pathways were powerfully activated, but did not seem to explain the impressive *IL-8 *response. There was marked up-regulation of *TP53BP2*, whose corresponding protein ASPP2 may interact with *H. pylori *CagA and cause marked p53 suppression of apoptosis. Other regulators of apoptosis also showed abberant regulation. We also identified up-regulation of several oncogenes and down-regulation of tumor suppressor genes as early as during the first 24 h of infection. *H. pylori *OMPLA phase variation did not seem to influence the inflammatory epithelial cell gene response in this experiment.

**Conclusion:**

In whole genome analysis of the epithelial response to *H. pylori *exposure, *IL-8 *demonstrated the most marked up-regulation, and was involved in many of the most important cellular response processes to the infection. There was dysregulation of apoptosis, tumor suppressor genes and oncogenes as early as in the first 24 h of *H. pylori *infection, which may represent early signs of gastric tumorigenesis. OMPLA^+/-^did not affect the acute inflammatory response to *H. pylori*.

## Background

*H. pylori *is well established as the primary cause of peptic ulcer disease and the initiator of the multistep cascade leading to gastric adenocarcinoma. Although gastric cancer is the fourth most common cancer worldwide and second only to lung cancer in causing cancer related deaths [1], there are remarkable differences in the distribution of this disease between western and eastern countries. The decrease in gastric cancer parallels *H. pylori *prevalence in the western world, but this phenomenon does not completely explain the great geographical differences in gastric cancer distribution. The reason why only 1-2% of *H. pylori*-infected individuals develop gastric malignancies remains unexplained, and includes both differences in bacterial strains, most importantly *cagA *status, host genetics and environmental aspects.

*H. pylori *carcinogenesis involves indirect action of the bacteria through chronic inflammation of the gastric corpus mucosa, and also direct action of *H. pylori *on epithelial cells. Persistent inflammation is associated with enhanced production of several pro-inflammatory cytokines, such as IL-1β, TNF-α, IL-6, IL-7 and IL-8 [2] which increase apoptosis, hyperproliferation and production of reactive oxygen and nitrogen species causing DNA damage and mutations. In addition, direct action of *H. pylori *on epithelial cells may also promote carcinogenesis. *cagA^+ ^H. pylori *strains inject bacterial products into epithelial cells through a sophisticated type IV injection process, which activates intracellular signaling pathways, in particular the mitogen-activated protein kinase family (MAPK) pathway [3] and nuclear factor kappa B (NF-κB), and may facilitate epithelial-mesenchymal transition [4], all of which may contribute to neoplastic transformation. Furthermore, tumor development is associated with proliferation and apoptosis inhibition [5,6], whereas excessive apoptosis is thought to promote gastric ulcer formation. The effect of *H. pylori *on gastric epithelial apoptosis has showed conflicting evidence. Several in vitro studies have showed that *H. pylori *stimulate apoptosis [7,8], whereas some in vivo studies demonstrate inhibition of apoptosis [9,10]. CagA injection into gastric epithelial cells up-regulates the anti-apoptotic MCL protein [11] and interferes with apoptosis-stimulating protein 2 of p53 (ASPP2) [12]. ASPP2 inhibition causes enhanced degradation of p53, in a way similar to DNA tumor viruses, thereby decreasing apoptotic activity, which may explain the increased risk of GC associated with *cagA^+ ^H. pylori *infection.

Tannæs et al. have previously reported that the *H. pylori pldA *gene, coding for bacterial outer membrane phospholipase A (OMPLA), displays phase variation resulting in 'ON' (OMPLA^+^) and 'OFF' (OMPLA^-^) switching of OMPLA activity due to a spontaneous slippage in a homopolymer (C) tract of the gene [13]. The OMPLA^+ ^variant was associated with increased bacterial survival in an acidic environment, adherence, hemolysis and release of urease and VacA compared to the OMPLA^- ^variant [14]. OMPLA has also been implicated in the virulence of other gastrointestinal pathogens [15], and a link between OMPLA activity and gastric inflammation has been suggested in a previous study [16].

Although the gastric epithelial cell response to *H. pylori *exposure has been subjected to many experiments since the discovery of the bacterium in 1984 [17], only a few studies have utilized cDNA microarray technology [18-29]. Almost all of these experiments have been performed on Asian *H. pylori *strains, and no authors have compared the epithelial cell response to OMPLA^+ ^against OMPLA^- ^bacteria. The aim of the current study was to investigate the temporal gene expression response of gastric epithelial cells exposed to a clinically obtained *H. pylori *strain, and to examine the contribution of OMPLA on the inflammatory response. Emphasis has been put on the most important biological responses using Gene Ontology (GO) terms and associated cellular signaling pathways.

## Results

**Figure 1:**
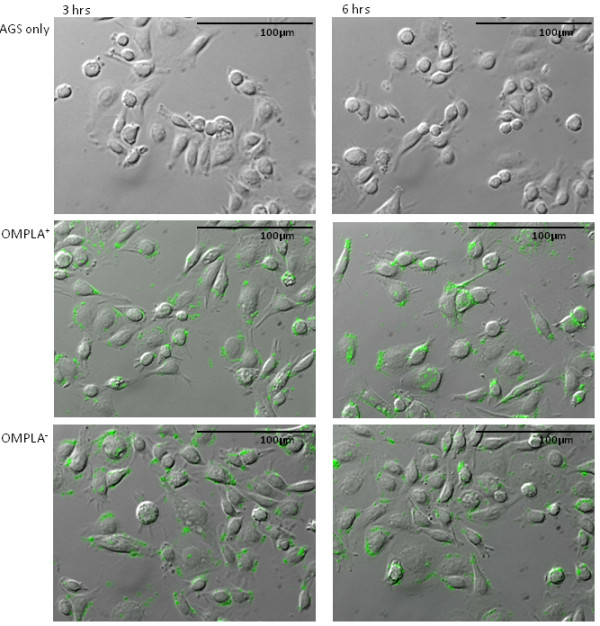
**Immunofluorescence images of AGS cells exposed to *H. pylori***. AGS cells were non-exposed, or exposed to OMPLA^+ ^and OMPLA^- ^*H. pylori *at a MOI of 300:1 and co-cultured for 3 and 6 h. The bacteria were stained with rabbit anti-*Helicobacter *antibody. Images were captured by fluorescent microscopy.

To study the cellular morphology following *H. pylori *infection at 3 and 6 h, non-exposed and *H. pylori *exposed cells were stained and examined with immunofluorescence microscopy (Figure 1). At both 3 and 6 h there was no significant difference in the ability between the OMPLA^+ ^and OMPLA^- ^*H. pylori *to adhere to AGS cells, and there were no significant differences in the morphological changes in the AGS cells in response to exposure to the two variants. We were not able to identify any statistically significant differences in the gene expression between the cells exposed to OMPLA^+ ^and OMPLA^- ^variants at any time point over the 24 h of co-culture (*p *< 0.05). We therefore concluded that analysis of the results could be performed without further consideration of differences in phase variation.

**Figure 2:**
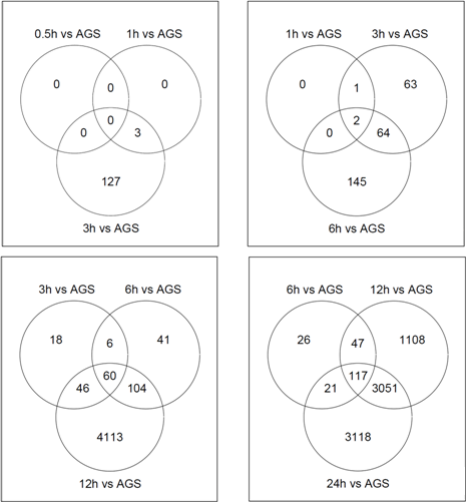
**Venn diagrams of significantly regulated genes**. Venn diagrams of differentially expressed genes of *H. pylori*-infected AGS cells compared to control cells (*p *< 0.05). The intersecting circles indicate overlapping genes at the indicated time points. AGS = non-infected control AGS cells.

The cDNA profile of *H. pylori *exposed AGS cells were compared against non-infected control cells at six separate time points within 24 h. 7498 chip probes corresponding to 6237 human genes showed differential expression in the infected cells compared to control cells at no less than 1 time point (*p *< 0. 05) (Additional file 1: Table S1). The number of significantly differentially expressed genes at each time point compared to non-infected AGS-cells, and how they overlap at different time points are illustrated in Table 1 and Figure 2.

**Table 1:** Number of differentially regulated genes

Time	0.5	1	3	6	12	24
**Up-regulated**	0	2	91	123	1679	2997

**Down-regulated**	0	1	26	65	2034	2492

**Total**	0	3	117	188	3713	5489

Number of significantly differentially regulated genes (*p *< 0.05) at each of the sampling time points after a period of co-incubation of *H. pylori *in AGS cells

**Figure 3:**
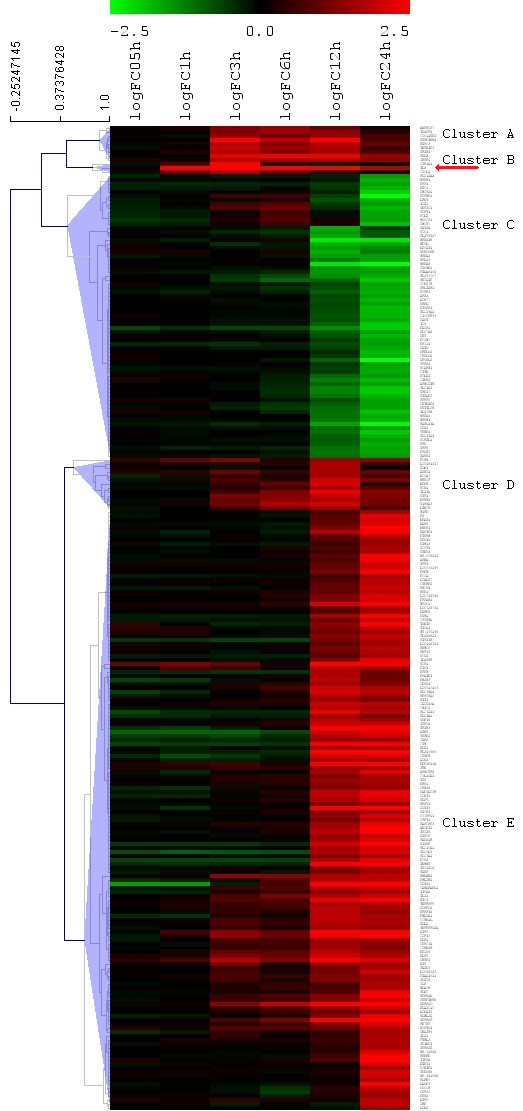
**Hiarchical clustering of the most significantly differentially regulated genes**. Hiarchical clustering of significantly differentially regulated genes (log_2_FC > 1.5, *p *< 0.05). Arrow points at *IL-8*.

There were no significantly expressed genes at 0.5 h, a moderate increase in the number of genes from 1 to 6 h, and a 20-fold increase from 6 to 24 h. From one sampling point to the next, most genes overlap, however a considerable number of unique genes were also differentially regulated at each time point (Figure 2). Approximately 47% of the total number of significantly expressed genes were up-regulated, and 53% showed down-regulation compared to control. Among the more than 6000 significantly expressed genes, *IL-8 *was the single most differentially expressed gene (Figure 3).

The list of all significant genes was analyzed for associated Kyoto Encyclopedia of Genes and Genomes (KEGG) signal pathways by Pathway Express at each time point. Significantly impacted pathways and corresponding Impact Factor (IF) are presented in Table 2. Early response signal pathways that were significantly affected included the epithelial cell signaling in *H. pylori *infection pathway, cytokine-cytokine receptor interaction, Toll-like receptor (TLR) signaling pathways as well as many cancer-related pathways and immunological pathways. At 1 h, *IL-8 *was involved in most of the affected signal pathways. At 3 and 6 h, most of the highest ranked pathways had several genes in common, such as *NFKB1, NFKB2, NFKBIA, NFKBIE, BIRC2, BIRC3, JUND, CCND1 *and *AKT3*. The phosphatidylinositol signaling system is assigned a high IF at 6 h due to the significance of one single gene, *PIK3C2B*, which is down-regulated by a log_2_FC of -0.58 and plays a key role in this pathway. At 12 h, the most affected cellular pathways were the leukocyte transendothelial migration, cell adhesion molecules, DNA replication pathway, p53 signaling pathway as well as several cancer-related pathways. Relatively similar results are seen at 24 h, however some of the cancer-related pathways are represented further down the list (data not shown, only top 10 shown in Table 2).

**Table 2:** Time course: KEGG cellular pathways and gene ontology

Time	KEGG cellular pathway name	IF	GO up-regulated genes	GO down-regulated genes
**0.5**	No significant genes		No significant genes	No significant genes

**1**	Epithelial cell signaling in Helicobacter pylori infection	16.6	No significant GO	No significant genes
	Cytokine-cytokine receptor interaction	8.1		
	Bladder cancer	7.5		
	Toll-like receptor signaling pathway	6.6		
	Base excision repair	6.0		
	Primary immunodeficiency	5.9		
	Pathways in cancer	5.4		

**3**	Epithelial cell signaling in Helicobacter pylori infection	17.8	anti-apoptosis	No significant GO
	Pathways in cancer	16.9	regulation of retroviral genome	
	Small cell lung cancer	14.2	replication	
	MAPK signaling pathway	14.2	T-helper 1 cell differentiation	
	Apoptosis	12.5	negative regulation of LPS-mediated signaling pathway	
	Adipocytokine signaling pathway	12.3	negative regulation of smooth muscle cell migration	
	Prostate cancer	11.4	regulation of MAP kinase activity chemotaxis	
	Toll-like receptor signaling pathway	11.1	protein amino acid dephosphorylation	
	T cell receptor signaling pathway	10.5	neutrophil activation	
	B cell receptor signaling pathway	9.9	entrainment of circadian clock	

**6**	Phosphatidylinositol signaling system	32.2	anti-apoptosis	No significant GO
	Epithelial cell signaling in Helicobacter pylori infection	15.5	regulation of retroviral genome	
	Small cell lung cancer	14.2	replication	
	Pathways in cancer	12.4	T-helper 1 cell differentiation	
	Apoptosis	11.6	neutrophil activation	
	Adipocytokine signaling pathway	10.1	negative regulation of I-kappaB	
	Toll-like receptor signaling pathway	8.9	kinase/NF-kB cascade	
	MAPK signaling pathway	8.7	induction of positive chemotaxis	
	Bladder cancer	8.5	myeloid dendritic cell differentiation	
	B cell receptor signaling pathway	8.3		

**12**	Leukocyte transendothelial migration	309.7	cell cycle arrest	response to unfolded protein
	Cell adhesion molecules (CAMs)	75.4	amino acid transport	S-adenosylmethionine biosynthetic process
	DNA replication	25.0	positive regulation of transcription	
	Cell cycle	20.0	response to stress	
	Pathways in cancer	19.4	regulation of MAP kinase activity	
	p53 signaling pathway	17.0		
	Antigen processing and presentation	15.7		
	MAPK signaling pathway	13.2		
	Small cell lung cancer	12.2		
	Circadian rhythm	11.9		

**24**	Leukocyte transendothelial migration	80.3	keratinocyte differentiation	cholesterol biosynthetic process
	Cell cycle	24.4	amino acid transport	response to unfolded protein
	p53 signaling pathway	20.9	keratinization	isoprenoid biosynthetic process
	Circadian rhythm	18.6	angiogenesis	creatine biosynthetic process
	DNA replication	18.0	apoptosis	response to oxidative stress
	Adherens junction	16.1	response to stress	
	Pathways in cancer	14.9	cell cycle arrest	
	Nucleotide excision repair	14.3	pyrimidine nucleotide metabolic	
	Ubiquitin mediated proteolysis	14.2	process	
	Phosphatidylinositol signaling system	13.7	induction of positive chemotaxis	

Significantly impacted KEGG cellular pathways and enriched Gene Ontology terms (biological processes only) (*p *< 0.05) at different time points following co-culture of *H. pylori *and AGS cells. Top 10 pathways/ontologies included where number exceeds 10. IF = impact factor

Because GO analysis simply associates differentially expressed genes with the ontologies, there is no attempt at ranking the true biological significance of individual genes or ontologies. Therefore, we included only genes with a log_2_FC > 1.5 in the GO analysis, excluding lesser significantly expressed genes that were likely to result in erroneous GO ranking. Only terms categorized under Biological Processes are included (Table 2), as these were the focus of the study. No GO terms were enriched at 0.5 or 1 h time points. Among the up-regulated genes at 3-6 h, the most frequently associated GOs were anti-apoptosis, and several inflammatory and anti-microbial processes such as regulation of retroviral genome replication, T-helper 1 cell differentiation, chemotaxis, neutrophil activation and immune activation. At 12-24 h, the up-regulated genes enriched ontologies like cell cycle arrest, apoptosis, stress response, amino acid transport, angiogenesis and keratinization, while certain biosynthetic processes are among the down-regulated terms.

Hierarchical clustering of the 245 genes with a log_2_FC > 1.5 formed 5 distinct clusters (A-E), at a distance threshold of 0.54, (Figure 3). Each cluster was examined for GO and cellular signal pathway associations (Table 3). GO analysis provided significant terms for all clusters (*p *< 0.05). Table 3 shows the top 10 significantly impacted cellular signaling pathways within each cluster, ranked according to IF. Cluster A contained 9 genes, and demonstrated steady levels at 6-12 h before showing a decline. Three genes were involved in anti-apoptotic processes and two genes were involved in MAPK signaling. Only 3 genes were assigned to cluster B, where there was a rapid and potent increase in expression during the first 3 h, followed by a decline. Of the 3 genes in the cluster, *IL-8 *and *CXCL2 *seemed to dictate many of the acute inflammatory processes like chemotaxis, immune response and neutrophil activation.

**Table 3:** Cluster profiling: KEGG cellular pathways and Gene Ontology

Temporal profile over 24 h	Cellular Pathway	Impact Factor	GO number	GO name
	MAPK signaling pathway	7.3	GO:0006916	anti-apoptosis
	Apoptosis	7.1	GO:0045063	T-helper 1 cell differentiation
			GO:0031665	negative regulation of LPS-mediated signaling pathway
			GO:0014912	negative regulation of smooth muscle cell migration
			GO:0043405	regulation of MAP kinase activity

	Epithelial cell signaling in *H. pylori *infection	12.4	GO:0006935	chemotaxis
	Cytokine-cytokine receptor interaction	10.2	GO:0006954	inflammatory response
	Bladder cancer	6.8	GO:0006955	immune response
	Toll-like receptor signaling pathway	5.9	GO:0045091	regulation of retroviral genome replication
	Pathways in cancer	4.8	GO:0042119	neutrophil activation
			GO:0050930	induction of positive chemotaxis
			GO:0030593	neutrophil chemotaxis
			GO:0030155	regulation of cell adhesion
			GO:0019722	calcium-mediated signaling

	Circadian rhythm	20.0	GO:0006915	apoptosis
	MAPK signaling pathway	10.7	GO:0006950	response to stress
	mTOR signaling pathway	7.5	GO:0007050	cell cycle arrest
	Tight junction	7.0	GO:0030216	keratinocyte differentiation
	Jak-STAT signaling pathway	6.7	GO:0006865	amino acid transport
	Cytokine-cytokine receptor interaction	6.5	GO:0031424	keratinization
	Regulation of autophagy	6.4	GO:0008652	amino acid biosynthetic process
	p53 signaling pathway	5.6	GO:0006220	pyrimidine nucleotide metabolic process
	Regulation of actin cytoskeleton	5.2		
	TGF-beta signaling pathway	5.2		
	Natural killer cell mediated cytotoxicity	4.7		

	Melanogenesis	8.3	GO:0030146	diuresis
	GnRH signaling pathway	7.6	GO:0030147	natriuresis
	ErbB signaling pathway	6.7	GO:0048661	positive regulation of smooth muscle cell proliferation
	Pathways in cancer	6.4	GO:0002268	follicular dendritic cell differentiation
	Epithelial cell signaling in *H. pylori *infection	5.7	GO:0031583	activation of phospholipase D activity by G-protein coupled receptor protein signaling
			GO:0014826	vein smooth muscle contraction
			GO:0002467	germinal center formation
			GO:0030578	PML body organization
			GO:0030195	negative regulation of blood coagulation
			GO:0043507	positive regulation of JUN kinase activity

	Antigen processing and presentation	13.7	GO:0006695	cholesterol biosynthetic process
	MAPK signaling pathway	9.7	GO:0006986	response to unfolded protein
	Bladder cancer	6.2	GO:0006916	anti-apoptosis
	Pathways in cancer	6.1	GO:0006139	nucleobase, -side, -tide and nucleic acid metabolic process
	Regulation of actin cytoskeleton	6.1	GO:0008299	isoprenoid biosynthetic process
			GO:0006601	creatine biosynthetic process
			GO:0009416	response to light stimulus
			GO:0043154	negative regulation of caspase activity
			GO:0007566	embryo implantation

Temporal profiles of 5 main clusters identified by hiarchical clustering of the 245 most differentially expressed genes (*p *< 0.05) and associated gene ontologies (biological processes only) and KEGG cellular signaling pathways in each cluster in *H. pylori *exposed AGS cells. Data points are at 0.5, 1, 3, 6, 12 and 24 h of co-incubation. Error bars represent ± standard deviation of expression within the cluster. Top 10 ontologies listed where number is exceeding 10

Cluster C comprised the largest cluster, and contained 150 genes that did not show any change until after 6-12 h. The GO terms apoptosis, cell cycle arrest and stress response genes were markedly enriched, and many of these genes such as *JUN, GADD45A, DDIT3, MKNK2, DUSP1, RPS6KA5, FLNC*, and *RASGRP *were also involved in MAPK signaling. Furthermore, *CSF2RA*, *IL24*, *IL20R *and the oncogene *PIM1 *were involved in Jak-STAT signaling and cytokine-cytokine signaling.

Cluster D showed a moderate increase peaking at 12 h, followed by a decrease towards 24 h. 13 genes were assigned to this cluster, including *EDN1*, one of the isoforms of the potent vasoconstrictor endothelin, which enriched virtually all of the listed GOs. *NFKB2*, one of two NF-κB subunits, *HBEGF *and *ETS1 *were also included in this cluster.

Cluster E demonstrated 71 genes that showed down-regulation after 6-12 h and included *FGFR3 *and several heat shock protein genes that were involved in the MAPK signaling pathway and apoptosis inhibition. Also, several GO biosynthetic processes were enriched.

**Figure 4:**
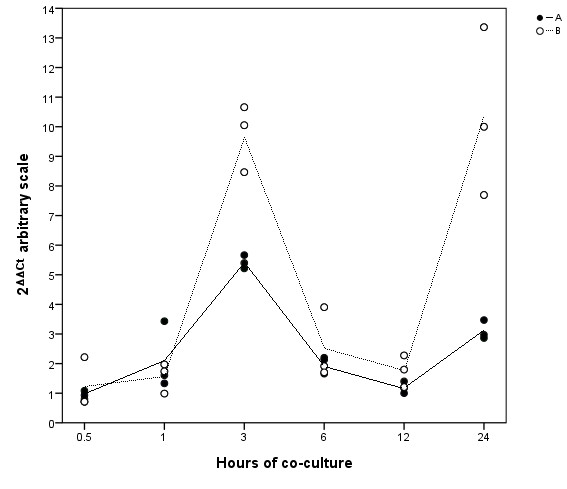
**Time-course of *IL-8 *mRNA expression in AGS cells co-cultured with *H. pylori***. Quantitative PCR analysis of *IL-8 *expression in *H. pylori*-infected AGS cells at six different sampling points over 24 h. Data points are the values of three cell culture replicates from two independent experiments, A and B. Lines represent the calculated mean within each of the experiments.

**Figure 5:**
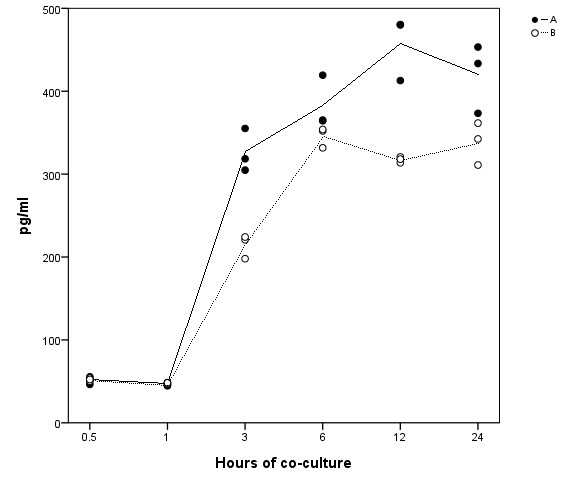
**Time-course of IL-8 protein expression in AGS cells co-cultured with *H. pylori***. ELISA analysis of IL-8 protein expression in *H. pylori*-infected AGS cells at six different sampling points over 24 h. Data points are the values of three cell culture replicates from two independent experiments, A and B. Lines represent the calculated mean within each of the experiments.

To confirm the microarray results, we chose to verify *IL-8*, as this was the single most differentially regulated gene in the study. mRNA and protein were sampled at the same time points and studied by rt-PCR and ELISA (Figures 4 and 5). There was an increase in *IL-8 *mRNA noticeable after 1 h and peaking at around 3 h. The *IL-8 *mRNA response then dropped towards 6 and 12 h. At 24 h there was a second increase, however with noteworthy variance between the two experiments. At 0.5 and 1 h of co-culture, IL-8 protein levels were low and did not show any change. Between 3 and 6 h of co-culture, there was a significant IL-8 increase which showed no further increase after 6 h.

**Figure 6:**
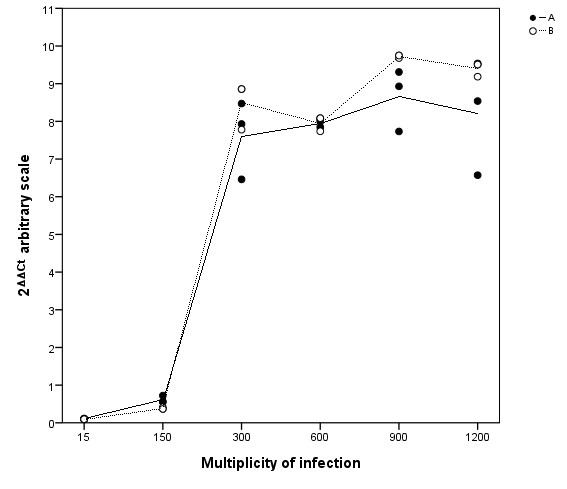
**Dose-response of *IL-8 *mRNA expression in AGS cells co-cultured with *H. pylori***. Quantitative PCR analysis of *IL-8 *expression in *H. pylori*-infected AGS cells, co-incubated for 3 h. Data points are the values of three cell culture replicates from two independent experiments, A and B. Lines represent the calculated mean within each of the experiments.

Lastly, we wanted to ascertain that the chosen MOI was stable with regard to AGS gene expression. We used *IL-8 *response as an indicator of gene expression, and AGS cells were co-incubated with *H. pylori *for 3 h at various MOI in two separate experiments (Figure 6). There was a modest *IL-8 *response at MOI 15:1 and 150:1, with a remarkable increase at MOI of 300:1. There were then negligible changes in *IL-8 *expression above 300:1, which suggested that the original inoculum of 300:1 was adequate to elicit a biological response without overloading the cell culture system.

## Discussion

In this study we demonstrate a significant, immediate response from AGS cells to the exposure to a *H. pylori *strain obtained from a clinical setting. More than 6000 human genes showed statistically significant differential regulation during the first 24 h of co-incubation.

*H. pylori *infection has been associated with both stimulation and inhibition of apoptosis. Some cell culture experiments demonstrate up-regulation of genes associated with apoptosis [7,8], whereas some in vivo studies demonstrate proliferation and apoptosis inhibition [9,10]. VacA toxin has been shown to cause apoptosis in several studies [30-33], whereas the role of CagA is conflicting. CagA has been associated with both stimulation and inhibition of apoptosis [11,12,34]. Biliary cells exposed to *cagA^+ ^H. pylori *at a very low inoculum (MOI 1:1) demonstrated increased cell growth, whereas at MOI of 200:1, apoptosis was stimulated [35]. CagA may even directly antagonize the pro-apoptotic effect of VacA, as seen in AGS cells [31]. Apoptosis occurs after a number of cellular events, leading to activation of caspase-3, which is thought to constitute the basic effector of apoptosis. In the present study, both inhibitory and stimulatory genes showed significant differential expression, demonstrating the complexity of the influence of *H. pylori *on apoptosis: caspase inhibitors *HSPA5 *and *DHCR24 *showed similar late down-regulation as heat shock genes *HSPA1B, HSPB1*, which are also associated with apoptosis stimulation (cluster E, Table 3). On the other hand, *TNFAIP3, BIRC2, BIRC3 *and *SERPINB2*, also associated with apoptosis inhibition, demonstrated early and persistent up-regulation grouped together in cluster A. However, positive regulators of apoptosis *PTPRH, TNFRSF12A, IL24, GADD45A, TRIB3, DDIT4, PHLDA4, PP1R15A *and *SQSTM1 *were all up-regulated in similar pattern after 6-12 h (cluster C). *MCL1*, an anti-apoptotic gene expressed in response to CagA injection [11], demonstrated increasing up-regulation over the course of the study. There were no significant changes in *BCL-2 *and very little increase in *BAX *expression in our study, two important genes that determine the sensitivity of cells to other apoptotic stimuli [36-39]. Noteworthy, there was marked up-regulation of *TP53BP2*, an important tumor suppressor gene (TSG) in human cancer, primarily stimulating p53 promotion of apoptosis genes. On the other hand, *TP53BP2 *is coding ASPP2 protein, which has also been shown to stimulate apoptosis independently of p53 [40-42]. However, Buti et al. recently demonstrated that CagA injected into gastric epithelial cells targeted ASPP2 protein to inhibit p53-mediated apoptosis [12]. The increased *TP53BP2 *expression seen in our study, might therefore potentiate this effect by increasing the CagA-ASPP2 interaction to cause increased inhibition of p53-mediated apoptosis. In fact, the current study showed that p53 target genes involved in apoptosis [43] such as *FAS*, *DR4*, *TNFRSF10B *(also referred to as *DR5/KILLER*), *DCR1*, *DCR2*, *P53AIP1*, *CASP6*, *APAF1 *and *BNIP3L *did not show any significant increase, and *BNIP3L*, *CASP6 *and *APAF1*, *BID *and *BAX *showed only little increase. p53 target genes regulating non-apoptotic cellular processes including *MDM2*, *GADD45A*, *CDKN1A *(also known as *P21 WAF1/CIP1*), *EGFR*, *CCND1*, *CCNG2 *and *TGFA *demonstrated moderate to marked up-regulation. This differential gene expression identified among the p53 target genes in this study, may indicate selective inhibition of p53-mediated apoptosis due to increased CagA-ASPP2 interaction, consistent with Buti's findings.

Nevertheless, this study was not designed to assess whether the overall sum of inhibitory and stimulatory signals facilitated apoptosis or proliferation of epithelial cells. The current results illustrate the complexity of apoptosis regulation in epithelial cells in response to *H. pylori *exposure, and the cluster analysis suggests that there is some biological coordination of gene expression regulating apoptosis. This may explain some of the complex carcinogenic mechanism of *H. pylori *in gastric adenocarcinoma. There is strong association between *H. pylori *infecton, in particular the *cagA*^+ ^genotype [44], and gastric adenocarcinoma [45,46], and also other cancers have been suggested to harbour a role for *H. pylori *[47,48]. Furthermore, the present study shows that several cancer-related KEGG pathways are impacted in AGS cells during 24 h of *cagA*^+ ^*H. pylori *infection, in particular pathways in cancer, bladder cancer, prostate cancer, small cell lung cancer and the MAPK pathway. Several individual oncogenes and cancer related genes were also increased during, and at the end of the study, including *ANGPT2, CEBPB, ECGF1, MMP7, MMP10, JUN, FOSB, EGFR, CTNNB1, ANXA1, CD55, CLDN1, KLK6, KRT7, LCN2, MYC, PIM1, PIM2, PIM3 *and *ATF3*.

*IL-8 *appears paramount in the acute inflammatory response to *H. pylori *infection, as this gene is involved in all significant response pathways in the initial cellular response to infection. Several authors have demonstrated increase in IL-8 in response to *H. pylori *in both in vivo [49] and in vitro [50,51] studies. IL-8 is a key chemokine in accumulating neutrophils. Gastric mucosal IL-8 levels have shown a positive correlation with the degree of stomach corpus inflammation [52], and IL-8 is also highly increased in gastric cancer [53,54]. Our findings are supported by other authors who have demonstrated that *IL-8 *mRNA in vitro peaks between 2 and 4 h before decreasing over the next hours under similar conditions [55,56]. Protein studies have shown steady state IL-8 levels after 3 h [50,57,58], which is also in harmony with our ELISA results, where marked IL-8 levels were detectable at 3 h and continuing to increase at 6 h before reaching a steady level. *H. pylori*-induced IL-8 secretion may be explained by both stimulation of the MAPK signaling system [59,60], and NF-κB activation through several pathways [61,62]. In the present study, MAPK signaling was ranked relatively high from 3 h onwards, based on IF calculations, and the cluster analysis showed that increasingly more genes in the MAPK pathway were affected after 6 h of *H. pylori *exposure. Regulators of NF-κB; *TNFAIP3*, *RELB *and *BIRC3*, which could also have explained the *IL-8 *expression, show increasing expression after 3 h (Additional file 1: Table S1), identical to the findings of Guillemin et al. [29]. Therefore, it is interesting that the great increase in *IL-8 *mRNA, which peaked as early as 3 h as shown in both the microarray and rt-PCR data, occurs before the onset of both MAPK and NF-κB signaling. The rt-PCR data, but not the microarray analysis, also demonstrated a second increase in *IL-8 *mRNA at 24 h, although with noteworthy variance between experiments. While it is possible that this second surge may be explained by MAPK and/or NF-κB activation, it is unlikely that MAPK or NF-κB signaling explain the initial, powerful *IL-8 *mRNA peak seen at 3 h. The present study is the first to demonstrate that among more than 38 000 human genes, *IL-8 *was the single most up-regulated gene by gastric epithelial cells in response to *H. pylori *exposure in vitro, and it appears feasible that mechanisms other than MAPK or NF-κB activation may be responsible for this up-regulation.

Although histopathological studies indicate that MOI around 10:1 appear in *H. pylori*-colonized gastric mucosa, laboratory conditions can never replicate the complex physiology of the human stomach. Much higher MOI have normally been used to study in vitro gastric epithelial cell response to *H. pylori *colonization, and MOI of 300:1 was our incoulum of choice, as we wanted a sufficient inoculum to induce a biological response from AGS cells, both at the mRNA and protein levels, as indicated by other experiments [35,63-71]. However, it is worth noting that in a recent report by Ritter et al., a marked IL-8 response from AGS cells exposed to *cagA^+ ^H. pylori *was seen at MOI ranging from 10:1 to 100:1 [61]. The IL-8 response was higher at MOI 100:1 compared to 10:1 in all the bacterial strains tested. The response to MOI 300:1 was not assessed. Neither *cagA *nor *vacA *status seemed to affect the IL-8 response at the higher inoculum. Ritter's study also showed that different cellular pathways were activated in response to high or low MOI. In some other studies, where non-gastric cells were exposed to *cagA*^+ ^*H. pylori*, low MOI was associated with apoptosis inhibition and cell growth, whereas high MOI stimulated apoptosis and inhibited survival [35,72,73]. Hence, the choice of MOI may be crucial for the study outcome. Nevertheless, based on our immunofluorescence studies, where we found sufficient bacterial adhesion to AGS cells, typical morphological changes, and most importantly, a marked IL-8 mRNA and protein response to MOI 300:1, we concluded that under our experimental conditions, 300:1 was adequate to elicit a biological response without overloading the system.

You et al. performed a similar microarray study published in 2010 [74], where AGS cells were exposed to *H. pylori *for 6 h. A relatively stable number of 300-400 genes were reported to be differentially expressed at each of the sample points, whereas our data showed a progressive increase in the number of genes from 0.5 to 24 h. In addition, key biological processes like chemotaxis, TLR signaling and epithelial cell signaling were reported as down-regulated. This is in contrast to our results, and also the findings of most other similar microarray studies [19-23,25,26,28,29,68,75], where these particular processes are regularly increased. However, many of these studies do indeed show somewhat conflicting results, possibly explained by differences in incubation conditions, bacterial strains and obsolete or proprietary cDNA arrays and technology.

We have previously suggested a potential role for OMPLA in inflammation [14,16]. OMPLA^+ ^variants were found to yield increased hemolysis, adherence and release of urease and VacA compared to the OMPLA^- ^variant. One of the aims of the present study was therefore to investigate the role of OMPLA on the gastric epithelial cell inflammatory response. We compared the gene expression profile of *H. pylori *OMPLA^+ ^exposed cells against OMPLA^- ^exposed cells at the 6 different time points. No significant difference was detected at any of the time points.

No other studies have directly investigated the role of OMPLA on the gastric epithelial cell inflammatory response, as the *pldA*/OMPLA status is unknown in most strains. Among the few full genome sequenced *H. pylori *strains, G27 carries a C7 repeat in the *pldA *gene [76] and B38 carries a C9 repeat, both giving rise to a truncated and inactive OMPLA [77]. Several experiments have demonstrated the ability of G27 to induce a significant IL-8 response [29,78], supporting our current observation that OMPLA^- ^*H. pylori *is indeed capable of inducing significant inflammation. One surprising result has been reported in a study of pH-regulated gene expression in the G27-strain [79], where Merrell et al. reported that *cagA *was consistently suppressed by low pH in *H. pylori *G27. Previous studies of other *H. pylori *strains, however, had suggested that *cagA *expression was induced at low pH.

Although the *pldA *phase variation did not appear to affect the inflammatory response in this study, phase variation of the *pldA *gene probably serves a purpose in other aspects of *H. pylori*. OMPLA activity is associated with increased survival at low pH [13,80]. The mechanism behind this property is not yet known. One possibility might be that OMPLA has adapted an as yet unknown function needed for this specific environment, in addition to phospholipase activity. Dorrell et al. have showed that a *pldA *knockout mutant was unable to colonize mice [81]. Salaün et al. have assessed changes in a spectrum of *H. pylori *phase-variable genes in a mouse model of gastric colonization [82]. *pldA *was among the most rapidly changing genes, with changes occurring within the first 3 days of colonization. The change in *pldA *showed a phenotypic selection from an initial inoculum which consisted of a mixture of ON and OFF phenotypes, to an exclusively ON population.

Wernegreen et al. have postulated that evolutionary selection will interrupt a slippery tract, such as the C-tract in the *pldA *gene, thus removing the possibility of phase variation [83]. When selection does not happen, the sequence feature must be to some benefit for the bacterium. It seems clear that the normal gastric environment is optimal for the OMPLA^+ ^phenotype, but for what niche the OMPLA^- ^phenotype is adaptive is currently unknown. One could speculate that the properties of the OMPLA^- ^variant could be useful when transferring from one human stomach to another.

## Conclusions

In summary, we have confirmed important biological processes and pathways affected by *H. pylori *infection of gastric epithelial cells described by many other authors. *IL-8 *was the single most differentially regulated gene among more than 38 000 genes tested, and seems fundamental in the epithelial cell reaction to *H. pylori *demonstrated by its involvement in the majority of the response processes that we have identified. Several intracellular signaling pathways are significantly impacted, such as the epithelial cell signaling in *H. pylori *infection pathway including the MAPK and NF-κB pathways, however none of these pathways seem to explain the very rapid up-regulation of *IL-8 *seen at 3 h. Furthermore, we have observed differential expression of both stimulatory and inhibitory apoptosis genes, suggesting dysregulation of apoptosis following *H. pylori *infection. Apoptotic p53 target genes showed little changes in regulation, whereas many non-apoptotic p53 target genes demonstrated a marked increase in expression. This phenomenon may be explained by selective inhibition of p53 caused by the ASPP2-CagA interaction.

Lastly, although gastric carcinogenesis is a very delayed consequence of *H. pylori *infection, we have seen up-regulation of cancer-related signaling, as well as aberrant regulation of oncogenes and TSGs as early as the first 24 h of infection.

The work presented in this study does not support the previous suggestion that OMPLA enzyme activity enhances inflammatory response induced by *H. pylori *in epithelial cells. However, the phase shift seen in the *pldA *gene probably plays a role in other aspects in the life of the bacterium.

## Methods

Human gastric epithelial cells were infected by the OMPLA^+ ^and OMPLA^- ^*H. pylori*, and mRNA and protein were sampled at 6 different time points within the first 24 h. The co-cultures were studied by immunofluorescent microscopy at 3 and 6 h to study bacterial adhesion and cell morphological changes. First, human whole genome cDNA microarray analysis was conducted to study gene expression changes in the *H. pylori-*exposed cells. Second, the epithelial cell response to the OMPLA^+ ^variant was compared against the OMPLA^- ^variant. Third, IL-8 levels were analyzed by real-time PCR and ELISA to verify the microarray results. Last, a dose-response experiment was performed to ensure adequate bacterial inocula.

### Bacterial strain and variants

The bacterial strain, *H. pylori *17B/RH, a representative isolate displaying *pldA *phase variation, was isolated from a non-ulcer dyspeptic patient referred to outpatient endoscopy and maintained at -70°C [13]. The two *pldA *phase variants gave rise to a functional phospholipase A (OMPLA^+^) and a truncated, non-functional phospholipase A (OMPLA^-^) respectively. The isogenicity of the variants was previously confirmed by amplified fragment length polymorphism [13]. The variants had the s1a/m2 *vacA *genotype and were *cagA *positive displaying an ABC EPIYA genotype [16,80]. The presense of the *cagα*, *cagβ*, *cagE*, *cagL*, *cagM*, *cagX *and *cagY *genes indicated that the variants harboured an intact *cag *pathogenicity island (*cag*PAI) and were capable of CagA translocation (unpublished data). Both variants displayed a truncated LPS.

The bacteria were cultured on blood agar plates under microaerobic conditions at 37°C for 48 h. After cultivation, the bacteria were harvested and suspended in phosphate buffered saline (PBS). Bacterial concentrations were estimated by measuring OD_600_. Aliquots of the OMPLA^+ ^and OMPLA^- ^bacterial suspensions were transferred to separate cell culture flasks at appropriate concentrations. Dilutions of the suspensions were also plated onto blood agar plates. After 5 days of microaerobic incubation, the colonies were counted and inspected for any OMPLA phase shifts.

### AGS cell line and inoculation of cell cultures

The gastric epithelial cell line AGS (American Type Culture Collection no: CRL 1739) was grown on RPMI supplemented with 2 mM L-glutamine and 10% foetal calf serum at 37°C in a CO_2 _incubator at a gas composition of 5% CO_2 _and 20% O_2_. When cells grew to a confluent monolayer of approximately 5,1 × 10^6 ^cells/flask (60%) the medium was changed to RPMI supplemented with 2 mM L-glutamine only. After an equilibration period of about 30 min, bacteria in PBS were added. To study AGS cell gene expression during the first 24 h, the cells were co-cultured with the *H. pylori *at a multiplicity of infection (MOI) of 300:1. The two phase variants (OMPLA^+ ^and OMPLA^-^) were assigned to separate co-cultures, to allow the investigation of the whole genome response to *H. pylori *infection *per se*, and also to study possible differences in the response to the OMPLA^+ ^and OMPLA^- ^variants. Co-cultured cells were incubated for 30 min, 1, 3, 6, 12 and 24 h, before RNA was stabilized by RNA*later *(Applied Biosystems, United States), and the cells were harvested.

To ensure that the obtained gene response was adequate, a dose-response experiment was performed, adding bacteria to AGS cells at a MOI of 15:1, 150:1, 300:1, 600:1, 900:1 and 1200:1. Cells were co-incubated for 3 h, before being immersed in RNA*later *followed by harvesting of the cells. Non-infected AGS cells served as a negative control. Both the time-course and the dose-response experiments were carried out in three cell culture replicates and independently performed twice on separate days.

### Microscopy and immunofluorescent staining

Briefly, the bacteria were added to AGS cells grown on glass coverslips at a MOI of 300:1. The cells were co-incubated for 3 and 6 h and then fixed by 4% formalin. Following blocking with 5% bovine serum albumin in PBS for 30 min, the bacteria were stained with rabbit anti-*Helicobacter *antibody (FITC, 1:200, ab30954, ABCAM PLC, USA) for 1 h at room temperature. Subsequently, the AGS cells were morphologically examined using a fluorescent microscope (Olympus IX81, Olympus, Japan) under a 40x objective.

### RNA isolation, quality control and cDNA synthesis

Total RNA was isolated using RNeasy Mini (Qiagen GmBH, Germany) according to the manufacturer's protocol. RNA concentration and quality were determined using a NanoDrop ND-1000 spectrophotometer (NanoDrop Technologies, USA) and Agilent 2100 Bioanalyzer (Agilent Technologies, USA). For real-time PCR, cDNA was prepared using a First-Strand cDNA Synthesis Kit (GE Healthcare, USA), according to standard protocol.

The Illumina TotalPrep RNA amplification Kit (Ambion Inc., USA) was used to amplify RNA for hybridization on Illumina BeadChips. To synthesize first strand cDNA by reverse transcription, we used total RNA from each sample collected above. Following the second strand cDNA synthesis and cDNA purification steps, the in vitro transcription to synthesize cRNA was prepared overnight for 12 h.

### Real-time PCR analysis

Each sample was tested in triplicate by real-time quantitative PCR (rt-PCR) on the 7900HT Fast Real-Time PCR system (Applied Biosystems). Expression of *IL-8 *was analyzed using custom *IL-8 *primer and probe (part no: 4331348, assay ID: Hs00174103_m1, Applied Biosystems). Mean cycle time (C_t_) was calculated, and the comparative C_t_-method [84] was utilized to control for background gene expression using reference gene GADPH (part no: 4333764F, Applied Biosystems).

### ELISA

IL-8 protein was measured in the cell culture supernatant by the Quantikine Human CXCL8/IL-8 enzyme linked immunosorbent assay (ELISA) kit, according to manufacturer's instructions (R&D Systems, USA). The test samples were not diluted. Serial dilutions of recombinant human IL-8 were used for standard curves. The optical density of the wells was determined using a microtitre plate reader (Varioskan, Thermo Scientific, USA) set to a wavelength of 450 nm, with wavelength correction set to 540 nm

### cDNA oligonucleotide microarray analysis

The gene expression profiles were measured using Illumina Human HT-12 v3 Expression BeadChip (Illumina, USA), which enables genome-wide expression analysis (48 800 transcripts, corresponding to approximately 37 800 genes) of 12 samples in parallel on a single microarray. 35967 of the probes were designed using the RefSeq (build 36.2, release 22) library and 12.837 probes were derived from the UniGene (build 199) database [85,86].

### Bioinformatics and statistics

R/BioConductor [87,88], with the package Beadarray [82], were used for preprocessing of the microarray text data from BeadStudio. Spatial artifacts were removed using BASH [89] before the expression data were log_2_-transformed and quantile normalized. Moderated t-tests [90] were then performed for each probe on the array to test whether the differential expression between the starting point and the later time points was significant. To account for multiple testing, adjusted *p*-values were calculated by controlling the false discovery rate (FDR), using the Benjamini-Hochberg procedure [91]. The differential expression was declared significant if the adjusted *p*-value (FDR q-value) < 0.05. The analysis was performed using the R statistical package [87] and the limma software package from Bioconductor [88].

To produce a reasonable sized list of the most differentially expressed genes, lesser expressed genes were filtered out. A cutoff level at log_2 _fold change (log_2_FC) > 1.5 was applied to the total genelist of 6237 significant genes (Additional file 1: Table S1), producing a list of the 245 most differentially expressed genes (Additional file 2: Table S2). For the selected genes, all 6 corresponding fold change values, including non-significant values, were assigned to the genelist for hierarchical clustering. Assuming that similarly expressed genes may share some of the same biological functions, the goal of hierarchical clustering is to group together genes with similar expression. In a time course study, it is most biologically relevant to cluster together genes that have a similar expression pattern, rather than expression magnitude. Consequently, the Pearson correlation coefficient was the appropriate distance measure in the clustering of our results.

Data were imported into Multi Experiment Viewer v 4.6.0 (MeV) software [92] for hierarchical clustering, and both non-clustered data and the clustered subsets were entered into Onto-Express and Pathway Express [93,94], part of the Onto-Tools software suite, for GO and KEGG signal pathway analysis. Pathway Express calculates an Impact Factor (IF) which is used to rank the affected pathways, based on the FC and the number of the involved genes, and the amount of perturbation of downstream genes [95].

The microarray data are available under the accession number E-MTAB-846 in the ArrayExpress database http://www.ebi.ac.uk/arrayexpress.

## Authors' contributions

LLE, YE and TMT performed inoculation and co-incubation of cells and bacteria, as well as performed ELISA and rt-PCR analysis. YE and TMT carried out immunofluorescence and microscopy. IRKB participated in the design of the study, and GB coordinated the study and helped to draft the manuscript. LLE carried out the microarray data analysis and wrote the main manuscript. All authors read and approved the final manuscript.

## Supplementary Material

Additional file 1 **Table S1**. The list of genes that showed significant differential expression at no less than 1 time point in *H. pylori *exposed AGS cells (*p *< 0.05). Click here for file

Additional file 2 **Table S2**. The list of genes that showed significant log_2 _fold change > 1.5 in *H. pylori *exposed AGS cells at no less than 1 time point (*p *< 0.05). Click here for file
